# Putting the theory before the data: is “massive modularity” a necessary foundation of evolutionary psychology?

**DOI:** 10.3389/fpsyg.2014.01158

**Published:** 2014-10-10

**Authors:** Ian D. Stephen

**Affiliations:** Department of Psychology and Evolutionary Psychology Research Group, Macquarie UniversitySydney, NSW, Australia

**Keywords:** massive modularity, evolutionary psychology, EEA, Santa Barbara school, human ethology

In this volume, Burke (2014) makes a number of arguments for why evolutionary approaches have failed to penetrate the rest of the field of psychology (what Burke refers to as “mainstream” psychology). While all of his arguments have merit, I will focus on one that I consider to be particularly important—the characterization by critics of the “Santa Barbara school” (Laland and Brown, 2011) as representative of all evolutionary approaches to psychology. Here, I agree with this point, and I expand upon Burke's point to argue that the focus on massive modularity as one of the foundational principles of evolutionary psychology is “putting the theory before the data,” and opens the discipline to criticism that is unwarranted for many of its researchers.

In 2013, I was fortunate to attend a talk by John Richer at the International Society for Human Ethology's Summer Institute, who argued that there is much to be gained from applying the ethological methodology of observation and documentation to clinical psychology settings. He was advocating deviating from hypothesis and experimentation, and applying a technique more akin to the production of ethnographies in social anthropology (Richer, 2014). While initially resistant to the idea, I later read Rozin's (2001) critique of the state of social psychological research. A comparison is made to Darwin's theory of evolution by natural selection which, he argues, was the result of a large body of observation, description and documentation that took place before the formalization of foundational principles. It grew out of an empiricist—as opposed to theorist—desire to understand the origins of species. Rozin argues that social psychology, in its rush to model itself on more established lines of research, such as biology and cognitive science, has skipped these important stages of observation and description, which he considers so critical to the development of a young discipline. In so doing, Rozin argues, social psychologists have rushed to formalize as theoretical underpinnings of the discipline ideas that have little supporting evidence, and have greatly restricted the range of acceptable topics for investigation within the field.

The situation in evolutionary psychology is similar, though not identical. Early in its conception, researchers attempted to formalize the field with a set of foundational principles—typically evolved psychological mechanisms, massive modularity of mind/domain specificity and the concept of an environment of evolutionary adaptedness, often assumed to be the Pleistocene (Cosmides and Tooby, 1987). These principles are not universally accepted within the community of researchers who take an evolutionary approach to psychology (Laland and Brown, 2011), and Burke (2014) argues that the massively modular view of the brain is not necessary for the application of evolutionary theory to psychology. Indeed, most research in the field is focused on gathering observations and testing hypotheses derived from fundamental evolutionary principles, rather than from the Santa Barbara school's formulation (Burke, 2014).

In most discussions about modularity and plasticity in the mind, the argument is really over the degree of modularity in the mind, and therefore the level on which selection operates. In much of the research being conducted in the field of evolutionary approaches to psychology, this distinction is, however, largely irrelevant. In contrast to the criticisms of many critics of evolutionary approaches to behavior (Benton, 2000), even adherents to the Santa Barbara school's formulation predict the evolution of flexible mental modules in order to allow flexibility of behavior in response to environmental and internal factors (Kurzban, 2002; see also Sperber, 2005). Consider the example of men's preferences for women's body size, which is hypothesized to represent a preference for healthy weight given the local environmental conditions. Men living in areas of food scarcity prefer higher BMI women, as this is most adaptive, while men in areas of food security prefer lower BMI women, as this is most adaptive given the local conditions (Tovée et al., 2006). This same hypothesis follows equally from a massive modularity, Santa Barbara school approach as from a mental plasticity, cultural evolution approach. The Santa Barbara school approach predicts that an evolved mental module for body size preference should have been selected to be sensitive to local ecological conditions, and would therefore predict the pattern of higher BMI preferences in areas of food scarcity and lower BMI preferences in areas of food security. A more moderate modularity approach would predict a mental module for attractiveness that can learn the appropriate preferences given the local ecological conditions, and thus predict the same pattern. Finally, a mental plasticity, cultural evolution approach would predict that culturally transmitted body size preferences would result in fitness benefits for those who carried the appropriate body size preference given the local ecological conditions, and would therefore predict the same pattern. It is not necessary to commit to one of these models of mind in order to formulate hypotheses based on evolutionary predictions (Burke, 2014). The necessary foundational principles are merely that behavior, cognition and perception have fitness consequences, and that selection shapes behavior, perception and cognition; something upon which all researchers adopting evolutionary approaches to psychology can surely agree.

While mainstream cognitive psychology and neuroscience are producing some convincing data that different types of information are processed in different brain regions—which could be considered modules—and there have been some well-reasoned defenses of the concept of massive modularity (e.g., Barrett and Kurzban, 2006), the conception of the massively modular mind lacks sufficient empirical evidence (Laland and Brown, 2011). Burke (2014) points out that, in the absence of alternative formally articulated sets of foundational principles, the Santa Barbara school's formulation presents critics of evolutionary approaches to psychology with a supposed foundational principle that lacks a solid empirical basis, and allows these critics to dismiss the entire field as built on shaky foundations. Ironically, the criticism that is aimed at evolutionary approaches to psychology (which, it should be noted, began well before the formalization of the Santa Barbara principles) provides substantial pressure to formalize that other disciplines did not have to weather during their early stages (Rozin, 2001). Laland and Brown (2011) point out that Wilson felt that sociobiology was held up to unfair standards: “While the sociological or cultural model is assumed to be true unless proven false beyond any possible doubt, the biological model is assumed to be false unless evidence is completely unassailable in their support.” This could also be said of evolutionary approaches to psychology more generally.

So where to from here? Others in this volume argue that the massive modularity of mind is an empirical question (Barrett et al., 2014), and I strongly agree. It may well turn out to be true, but before identifying this model of mind as a foundational principle, it is important to ensure that it is well supported empirically. In the meantime, it is encouraging to note that the early attempt to formalize foundational principles has not led to the over-focus on a small number of topics and techniques that Rozin (2001) decries in social psychology. Evolutionary approaches to psychology investigate a wide range of topics, from mate selection, life history strategy, food gathering and sharing, cooperation and altruism, aggression, gender roles, and parenting, and use a wide range of techniques—experimental psychological techniques, game theory, ethology, and ethnographic observations to name but a few. Anyone who attends HBES, EHBEA, ISHE or any of the other conferences dedicated to the approach will discover that new fields of study are constantly being approached through the lens of evolutionary theory.

In conclusion, then, while there is pressure from critics of the field to declare a set of foundational principles for the field, including determining whether or not the mind is domain specific and massively modular, these are empirical questions that require further research. Further, the structure of the mind is not a prerequisite for the investigation of psychology through an evolutionary lens. The field should therefore continue to research the question of modularity of mind, and continue to explore the broad range of human behavior and cognition through observation, documentation and hypothesis generation and testing. There is little to be gained by prematurely formalizing the foundations of the field—putting the theory before the data—particularly if those foundations later turn out to be shaky.

## Conflict of interest statement

The author declares that the research was conducted in the absence of any commercial or financial relationships that could be construed as a potential conflict of interest.
